# Psychological Mediation of Dysfunction and Hyperfunction of Respiratory Regulation

**DOI:** 10.3390/bs10010005

**Published:** 2019-12-19

**Authors:** Julia Koniukhovskaia, Elena Pervichko

**Affiliations:** Faculty of Psychology, Lomonosov Moscow State University, 125009 Moscow, Russia; elena_pervichko@mail.ru

**Keywords:** respiratory mechanics, hyperventilation, breath holding, diving, anxiety, self-control, clinical psychology

## Abstract

This research investigates the continuum between the dysfunction and the hyperfunction of breath regulation and presents the psychological mediation that supports or disrupts this regulation. The pilot study compared breathing regulation in patients with hyperventilation syndrome (HVS), free divers, and healthy volunteers. To examine the ability of voluntary respiration regulation, breath holding involving “easy-going” and “struggling” phases was used. Psychological mediation was assessed through (a) respiratory experience interviews, (b) anxiety levels, and (c) psycho-semantic techniques. Free divers have a bigger “positive breathing vocabulary” and can endure the conflict between the physiological need to inhale and the voluntary motivation to hold their breath for longer. The connection between emotions and negative breathing experience in patients with HVS leads to less breathing control.

## 1. Introduction

Breathing is the most important body function that inconspicuously adapts to physiological needs and supports homeostasis at each moment [1]. It is generally known that respiration regulation is both an autonomous and a voluntary function. Despite the fact that everyone learns to regulate breathing for speech, respiration regulation has been eluding psychological research.

The question of how psychological factors influence respiration regulation is particularly important for free diving, i.e., diving which relies on breath holding. At the moment, the world record for breath holding in men is 11 min and 35 s, set by Stéphan Mifsud in 2009, and the deepest dive in one breath hold is 214 m, made by Herbert Nitsch in 2007 [2].

These world records have given rise to physiological studies that reveal patterns of adaptation during breath holding, such as mammalian dive response [3,4]. In recent decades, an interest in the neuropsychological consequences of prolonged breath holding [5,6] and the personal characteristics of free divers has arisen [7,8].

But how do free divers become aware of breathing? Does it help to regulate it voluntarily?

The awareness of internal sensation during a dive is important because only the inner feelings prompt the free diver to float up when necessary, due to the risk to health and life [9].

Opposite to remarkable breath regulation in free diving, there are breathing dysfunctions resulting from the hyperventilation syndrome (HVS). HVS is a pathological pattern of breathing, in which the increase in pulmonary ventilation is inadequate for the functional needs of the body [10]. Over breathing causes respiratory alkalosis and, as a result, provokes respiratory, cardiovascular, gastrointestinal, muscular, neurologic, and psychological dysfunctions [11]. The etiologic reasons for HVS may be pathological, biomechanical, biochemical, environmental, habitual, psychological, or a combination. HVS may aggravate the underlying cause of the disease or create medically unexplained symptoms due to the psychological factor. 

Because of the close relationship between breathing and emotions [12], HVS is a frequent satellite of generalized anxiety disorder, panic disorder, phobias, and posttraumatic stress disorder [13,14,15]. Anxiety can raise breathing frequency and dyspnea, but the shortness of breath may trigger anxious arousal [16]. It forms a vicious circle and increases the symptoms of HVS [17]. When hyperventilation unexpectedly arises, it initiates a panic attack. The diffuse anxiety during an HVS attack may be transformed into different fears and phobias [17]. HVS also accompanies posttraumatic stress disorder that is explained by the developed conditioned reflex between stress stimulus and the frequency of breathing [18].

Fear is not an inevitable reaction to HVS [19]. The interpretation of internal feelings during dyspnea plays an important role [20]: The patients may see HVS as a harbinger of the imminent threat. Numerous experimental studies of dyspnea provocation have shown variation in individual responses to fear of suffocation [21]. A more negative interpretation is associated with the activation of defensive reactions and panic attacks in response to breathing with increasing resistance [22,23].

It is difficult to estimate the prevalence of HVS, as there are no uniform evaluation standards or differential diagnosis procedures. However, 8% of the general population, 10% of patients in general practice, and 29% of patients with asthma suffer from HVS [24]. So, one in ten adults may be experiencing potentially threatening symptoms of HVS and the impairment of their life quality. This fact justifies the relevance of the HVS study.

The comparison of breathing regulation in free divers and HVS patients raises the question of differences in the awareness of breathing in these cases. Why can some people dive tens of meters and others suffocate in the subway?

To answer this question, our research relied on the cultural–historical approach [25,26] and the psychology of corporeality [27,28]. According to the first approach, the human body, in the same way as the psyche, changes through cultural “transmission”. Through this process, the corporeality obtains the following traits of higher mental functions (HMFs): It becomes socially acquired, mediated by social meanings, and voluntarily controlled. Mediation in this approach is the use of signs and symbols for the awareness and the regulation of behavior. First sign-symbolic mediation is used in interpersonal communication, which then turns into internal mental tools for self-regulation.

The psychology of corporeality allows us to describe the normal and abnormal psychosomatic development of a person through the same psychological laws and mechanisms [29]. It also makes it possible to distinguish between psychosomatic phenomena of normal development and the psychosomatic symptoms in pathology. Thus, free diving is referred to as an example of normal psychosomatic development and HVS as a psychosomatic symptom. 

The research aims to show the role of psychological mediation in hyper-functional and dysfunctional respiration regulation among free divers and patients with hyperventilation syndrome. 

## 2. Materials and Methods

### 2.1. Participants

This paper presents the results of a pilot study in which 60 participants divided into three groups were involved:

(1) 20 certified free diving instructors from the Russian Freediving Federation (10 men and 10 women at an average age of 37.6 ± 10.5 years).
(2) 20 healthy volunteers (10 men and 10 women at an average age of 35 ± 11.5 years).
(3) 20 patients with neurogenic hyperventilation (15 women and five men at an average age of 42.5 ± 12.9 years).

The patients were recommended for the research by doctors (pulmonologist, neurologist, general therapist, or psychiatrist) and had no concomitant respiratory disorders. In addition to chronic HVS, 10 patients suffered from regular panic attacks, eight patients experienced a panic attack in the past, and two patients had not experienced any panic attacks. The ethics of research at our university evaluates meeting of the Academic Council of the Faculty of Psychology at Lomonosov Moscow State University, which approved the study protocol (№11, 28.12.2018).

### 2.2. The Procedure

The research team designed a four-stage diagnostic procedure.

The first stage consisted of an interview about respiratory experience, which included questions about psychosomatic predisposition (respiratory disorders in themselves and relatives, psychological trauma associated with the breathing function, panic attacks, etc.) and experience in breathing exercises (meditation, diving, swimming, etc.).

In the second stage, the Nijmegen Questionnaire [30] for the diagnostics of the hyperventilation symptoms and the State–Trait Anxiety Inventory (STAI) [31] were used.

The third stage involved the psycho-semantic technique “Classification of sensations” [27,32]. The original version of the technique consists of 80 cards, from which the volunteer is asked to choose words describing familiar, meaningful, painful, life-threatening, and often painful sensations. For the purpose of this research, the technique was modified: The participants were to choose words describing breathing experience; its negative and positive aspects. The “vocabulary lists” of each stage were calculated as a percentage of all the familiar words to describe the inner experience. To conduct the qualitative analysis of “vocabulary” at each stage, all words were classified by modality (physical, emotional, or intermediate), valence (pleasant, unpleasant, intermediate), and the frequency of use (often, medium, and rarely) based on the data from additional research [33].

At the fourth stage, the participants were asked to hold their breath for as long they could (four measurements). Three parameters were taken into consideration: The time of the initial “easy-going” phase before the urge to inhale, the “struggling” phase during the strong need to inhale, and the total time of the breath-hold. The difference between the “easy-going” and “struggling” stages was studied based on the participants’ self-reports and their non-verbal signal demonstrating increasing respiratory discomfort. Between the measurements, each subject rested longer than their prior breath hold. Four measurements of breath holding were made because of the increasing adaptation of mammalian diving response during retests [34].

### 2.3. Data Analyses

The results were processed through statistical software SPSS for Windows, version 22.0 (Copyright SPSS Inc., Chicago, IL, USA, 2014). Methods of descriptive statistics, correlation, and variance analysis (ANOVA) were used.

## 3. Results

### Breath Holding (BH)

**Figure 1:**
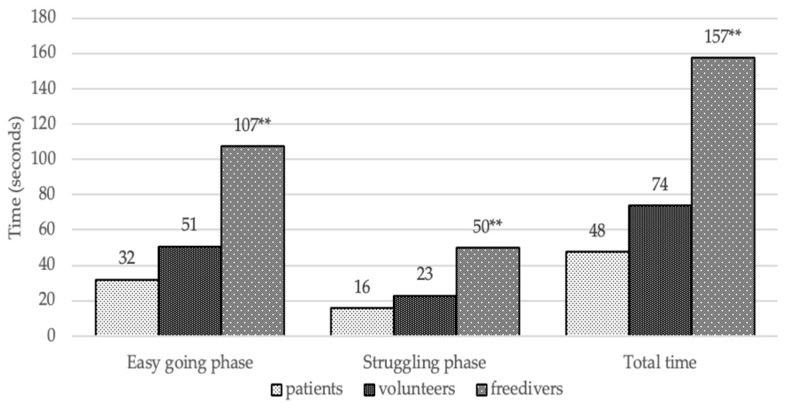
The average duration of breath hold parameters. Conventional sign: * The significant differences are *p* < 0.05, ** the significant differences are *p* < 0.005.

Figure 1 shows that free divers had a twice-as-long duration of breath hold than the volunteers (*p* < 0.001) and the HVS patients (*p* < 0.001). Free divers also demonstrated the longest ability to cope with the struggling phase, in comparison with the two other groups (*p* < 0.001). This parameter reflected the conflict between the vital need to breathe and the voluntary motive for a long breath hold. At the same time, the patients with HVS had the shortest breath hold and lowest ability to cope with the unpleasant sensation during the test. 

**Figure 2:**
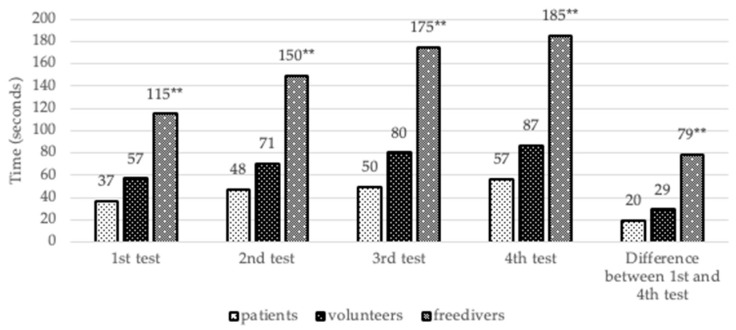
Durations of breath holding from first to fourth tests. Conventional sign: * The significant differences are *p* < 0.05, ** the significant differences are *p* < 0.005.

Comparison analysis of first and fourth retest revealed that free divers had a significant lengthening of breath holds after retests compared to healthy volunteers (*p* < 0.001) and patients (*p* < 0.001), which could indirectly reflect bigger physiological adaptation, such as mammalian dive response [3,4] (Figure 2). 

Self-reports after the breath hold demonstrated that 65% of free divers experienced breath holding (BH) neutrally, and in 30% of cases they even experienced pleasure. Only 45% of patients experienced BH neutrally, while in 55% of patients, BH caused symptoms of HVS (heaviness in the chest, burning behind the sternum, cough, etc.), increased anxiety, or memories of emotionally significant situations. In the control group, 70% of participants experienced a neutral reaction; 15% had anxiety, and another 15% felt better (*p* = 0.001, Pearson’s chi-squared test). 

The interview about the respiratory experience revealed that the patients had more chronic disorders and smoking habits in comparison to the healthy volunteers (*p* = 0.002, *p* = 0.046) and free divers (*p* < 0.001, *p* = 0.046). HVS patients also had more fears than the volunteers (*p* = 0.004). It was also found that patients witnessed respiratory diseases in their relatives more often than free divers (*p* = 0.059). These results suggested that the presence of chronic diseases and the identification of relatives with respiratory disorders could contribute to the rise of panic attacks: Patients perceive sensations in hyperventilation as a manifestation of another disease.

What was more, the free divers significantly exceed other participants in how often they lost consciousness during BH (*p* < 0.001 for both groups) and observed respiratory function accidents (*p* = 0.001 for both groups). The last fact did not cause free divers to have a negative fixation on the breathing function, unlike patients with HVS. It was also found that, in addition to BH, free divers practiced a variety of breathing regulation techniques (breathing exercises, meditation, swimming), whereas the differences between the control group and patients were not revealed.

**Figure 3:**
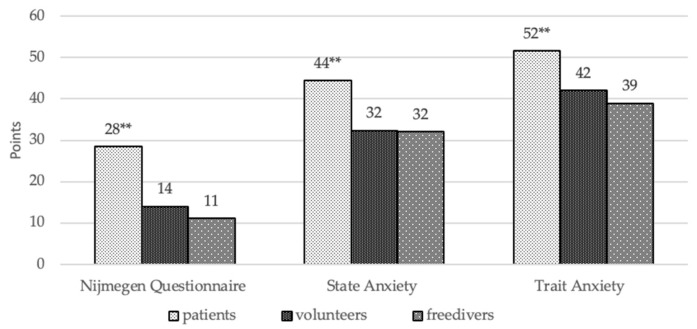
The results of the Nijmegen Questionnaire and State–Trait Anxiety Inventory (STAI). Conventional sign: * The significant differences are *p* < 0.05, ** the significant differences are *p* < 0.005.

The results of the Nijmegen Questionnaire can be seen in Figure 3, which shows that the patients demonstrated higher levels of hyperventilation symptoms than the volunteers (*p* < 0.001) and the free divers (*p* < 0.001). The comparison of the groups to the State–Trait Anxiety Inventory also revealed that patients had a significantly higher level of both types of anxiety in comparison with the free divers (*p* = 0.001) and the control group (*p* = 0.001); the difference between the free divers and the healthy individuals was not found. 

There was a strong negative correlation between all parameters of BH to HVS and both types of anxiety (Table 1).

**Figure 4:**
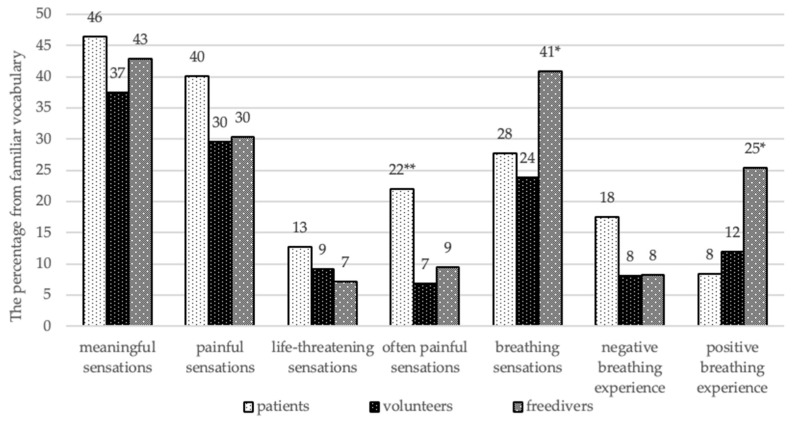
The volumes of vocabulary in the classification of sensations (%). Conventional sign: * The significant differences are *p* < 0.05, ** the significant differences are *p* < 0.005.

Quantitative analysis of mediation of internal sensations showed (Figure 4) that HVS patients had a larger vocabulary of “often painful” sensations than free divers (*p* = 0.001) and the control group (*p* < 0.001). Compared with free divers (*p* = 0.006) and healthy individuals (*p* = 0.005), patients also had the expanded vocabulary of “negative breathing experience”, which contained the same words from the vocabulary lists of “painful”, “life-threatening”, or “frequent painful” sensation. At the same time, free divers had significantly bigger “breathing vocabulary” in comparison with the control group (*p* = 0.007) and the patients (*p* = 0.046), which indicated a greater mediation of respiratory function. This expansion was provided by the volume of “pleasant breathing sensations”, which is twice that of patients (*p* = 0.003) and the control group (*p* = 0.022).

The correlation analysis showed that the vocabulary of “frequent painful” sensations had a significant negative association with the duration of BH (*p* = 0.011) and the “struggle phase” (*p* = 0.012). That means that those having many painful sensations could not long withstand the motivational conflict that occurred at the stage of the struggle between vital needs to inhale and voluntary motivation for holding their breath. The negative correlation between the raising from first to fourth tests and the volume of “frequent painful” sensations (*p* = 0.034) suggested that the experience of a large number of unpleasant and painful sensations led to less effective physiological reflex adaptation to hypoxia.

The volume of positive breathing experiences had a strong positive correlation with all parameters of BH: The “easy-going” phase (*p* = 0.002), the “struggling” phase (*p* = 0.001), the duration of BH (*p* < 0.001), and the difference between the first and fourth tests (*p* = 0.04). This showed that the association of breathing with pleasant experiences not only lengthened the stage of “motivational struggle”, but also postponed its occurrence, which, in general, aids longer breath retention.

The qualitative analysis of vocabularies showed that the “frequent painful” vocabulary of the patients dominated in emotional descriptors, in comparison with healthy individuals (*p* = 0.003) and free divers (*p* = 0.049). Free divers’ breathing vocabulary had more intermediate body-emotion words (*p* = 0.002), words with positive valency (*p* < 0.001), and frequently used word-descriptors *(p* = 0.013), in comparison with the HVS patients. There was also found a change of conventional meaning of the words in unpleasant breathing vocabulary in patients, as they described it through using words with positive connotations.

## 4. Discussion

The study showed that free divers, who often practiced breath holds, demonstrated broader “breathing vocabulary” due to a higher frequency of words describing pleasant sensations. This could mean that they rethought the respiratory discomfort that arose at the “struggle” stage during diving, while people without proper training saw the “struggle stage” as “agony”, and it was predominantly represented by unpleasant “respiratory vocabulary”.

The typical correlation of pleasant respiratory sensations with the ability to hold their breath longer in free divers indicated that the traditional sports approach of “overcoming oneself” when teaching free diving is not effective and not possible. First, the technique of “overcoming” the unpleasant sensations can lead to the loss of consciousness and life threat. Secondly, it is the ability to enjoy the process and to find pleasant feelings in extreme breathing experience while diving that makes it possible to extend the breath hold.

Identified during the psycho-semantic research, the contribution of psychological mediation to the breathing regulation marked the importance of not only the physiological adaptation of the free diver (for example, hypoxia tolerance), but also of the psychological characteristics of an athlete, i.e., his ideas about breathing and the ability to name the body sensations when learning to free dive.

The analysis of the “significant” and the “respiratory” dictionaries of patients with HVS revealed their fixation on unpleasant sensations. When naming “frequent painful” sensations, they more often referred to emotional rather than physical sufferings, which distinguished them completely from healthy people and free divers. Descriptors, which mean pleasant sensations in the standardization sample, reflected negative respiratory experience in HVS patients, which could be interpreted as a distortion of the conventional meanings of words.

Mixing emotions with painful sensations showed that patients with HVS had distorted sign-symbolic mediation of emotions and physical phenomena. The problem of differentiation between bodily sensations and emotions leads to the fact that patients see the physiological changes accompanying negative emotions in stressful situations as a disease and consult a doctor to cure it. At the same time, patients are not aware of the context in which the emotions appear. These data allow us to suppose that personal meanings and personal interpretations of the respiratory sensations can become an important “target” of psychotherapy.

The cultural–historical approach was used to explore respiration regulation for the first time in this pilot study. As such, our exploratory research had some limitations. Firstly, the number of participants was only 60 people across three groups. It is necessary to continue research involving more participants. Secondly, further study of psychological mediation of voluntary breathing regulation seems promising, taking into account biofeedback in physiological parameters such as cardiovascular response, respiratory movements, level of blood oxygenation, and gas composition at breath hold. This direction of study will allow correlating real physiological changes with the perception of bodily processes during breath hold, which can later be applied as a method of biological feedback for the treatment of functional respiratory disorders.

Third, an important step in the study may be a comparison of various theoretical approaches in the study of mediation of respiration regulation. The inability to distinguish between emotions and body sensations revealed in our research can be studied in terms of alexithymia which is characterized by difficulties in identifying and describing one’s own emotions [35]. Enough studies about the relationship between alexithymia and HVS have not been identified. Consequently, this may form the basis for further research. The question of personal interpretations of respiratory sensations during HVS still remains open and seems a promising research question. 

## 5. Conclusions

The data confirmed the mediated nature of respiratory function and the possibility of voluntary regulation of this function in accordance with the theory and methodology of the cultural–historical theory of mental development or “Vygotsky–Luria–Leontiev School” [25,26,36] and psychology of corporeality [27]. It is necessary to continue the study of the meaning of respiratory sensations in hyperventilation syndrome and to test the hypothesis about the interpretation of internal sensations, which can be a “target” during a psychological correction. Attention to the personal meaning of respiratory sensations during breath holds could be helpful to teach free diving and improve athletic performance.

## Availability of Data and Materials

The datasets used and analyzed during the current study are available from the corresponding author on reasonable request. 

## Figures and Tables

**Table 1:** Correlation analysis (Spearman rank correlation) between breath hold (BH), Nijmegen Questionnaire, and State–Trait Anxiety Inventory (N = 60).

		Easy Going Phase	Struggling Phase	Total Duration of BH	Difference between First and Fourth Test
Nijmegen Questionnaire	Correlation coefficient	−0.374	−0.419	−0.424	−0.448
	Bilateral significance	0.003	0.001	0.001	0.000
State Anxiety	Correlation coefficient	−0.329	−0.349	−0.392	−0.354
	Bilateral significance	0.010	0.006	0.002	0.006
Trait Anxiety	Correlation coefficient	−0.350	−0.456	−0.440	−0.304
	Bilateral significance	0.006	0.000	0.000	0.018
